# Predictive model for the intraoperative unresectability of hilar cholangiocarcinoma: Reducing futile surgical exploration

**DOI:** 10.1371/journal.pone.0258522

**Published:** 2022-04-13

**Authors:** Jinglin Song, Xiaofeng Lei, Heng Lin, Haisu Dai, Xingchao Liu, Yan Jiang, Feng Hu, Yuancheng Li, Haining Fan, Leida Zhang, Zhiyu Chen, Chengcheng Zhang

**Affiliations:** 1 Department of Public Economic System and Policy, School of Public Administration, Southwestern University of Finance and Economics, Chengdu, Sichuan, China; 2 Department of Hepatobiliary Surgery, Liaocheng People’s Hospital, Liaocheng, Shandong, China; 3 Department of Hepatobiliary Surgery, Southwest Hospital, Third Military Medical University (Army Medical University), Chongqing, China; 4 Sichuan Academy of Medical Sciences & Sichuan Provincial People’s Hospital, Chengdu, Sichuan, China; 5 College of Basic Medical Sciences, Third Military Medical University (Army Medical University), Chongqing, China; 6 Qinghai University Affiliated Hospital, Xining, Qinghai, China; MD Anderson Cancer Center, UNITED STATES

## Abstract

**Introduction:**

Surgical exploration is widely performed in hilar cholangiocarcinoma (HCCA), but the intraoperative resectability rate is only 60%-80%. Exploration substantially increases pain and mental stress, and the costs and length of hospital stay are considerably increased. Identifying preoperative risk factors associated with unresectability could decrease unnecessary exploration.

**Materials and methods:**

In total, 440 HCCA patients from multiple centers were enrolled. Those receiving surgical exploration were divided into the resected and unresected groups. Morphological variables including Bismuth classification, lymph node metastasis and vessel invasion were obtained from radiological exams. Logistic regression for the training cohort was used to identify risk factors for unresectability, and a nomogram was constructed to calculate the unresectability rate. A calibration curve assessed the power of the nomogram.

**Results:**

Among 311 patients receiving surgical exploration, 45 (14.7%) were unresectable by intraoperative judgment. Compared with the resected group, unresected patients had similar costs (p = 0.359) and lengths of hospital stay (p = 0.439). Multivariable logistic regression of the training cohort (235 patients) revealed that CA125, Bismuth-Corlette type IV, lymph node metastasis and hepatic artery invasion were risk factors for unresectability. Liver atrophy (p = 0.374) and portal vein invasion (p = 0.114) were not risk factors. The nomogram was constructed based on the risk factors. The concordance index (C-index) values of the calibration curve for predicting the unresectability rate of the training and validation (76 patients) cohorts were 0.900 (95% CI, 0.835–0.966) and 0.829 (95% CI, 0.546–0.902), respectively.

**Conclusion:**

Analysis of preoperative factors could reveal intraoperative unresectability and reduce futile surgical explorations, ultimately benefiting HCCA patients.

## Introduction

Tumor resection is the preferred treatment for hilar cholangiocarcinoma (HCCA) with vastly improved survival outcomes [1]. The prognosis of HCCA is poor because of inherent tumor malignancy and a high unresectability rate [2]. Surgical exploration is widely performed, but the total resection rate of HCCA is only approximately 50% [3–6]. Regardless of increased costs and a prolonged length of stay (LOS), surgery could lead to pain and mental stress in patients. With the development of preoperative testing tools, unresectability can be satisfactorily assessed with improved accuracy, while exploration, such as staging laparoscopy, can be reduced [7]. Establishing a preoperative model that evaluates resectability can reduce the number of futile explorations and benefit patients.

Resectability is highly correlated with pathological features. Because the tumor is located in the first porta hepatis, vascular involvement is common in HCCA. Because of the biological characteristics of HCCA, lymph node metastasis is often observed. Tumor staging systems such as the Bismuth-Corlette (BC) classification [8] and Memorial Sloan Kettering Cancer Center (MSKCC) system [5] mainly stage these tumors considering bile duct invasion, vascular invasion and lymph node metastasis. These variables are highly correlated with HCCA intraoperative unresectability [1, 6]. Additionally, variables based on blood parameters, such as tumor biomarkers, are associated with the resectability of HCCA [9]. CA19-9, CA125 and CEA are indicators of unresectability. These parameters expand the prediction criteria of HCCA. With the development of radiological examinations, the accuracy of predicting resectability has been drastically increased [3, 10, 11]. High-resolution CT and MRI with specific sequences can optimize unresectability evaluations. Radiomics also assists in the prediction of vital variables such as lymph node metastasis [11]. Because of improved surgical skills and preoperative examinations, complex resection can be performed safely, and the resectability rate has increased in recent years [7]. Because the resection rate of HCCA has improved over time, the risk factors for intraoperative unresectability may also vary. Thus, reanalysis of the preoperative factors associated with intraoperative unresectability may assist in reducing the number of futile explorations and improve clinical practice.

This study analyzed multicenter data and proposed variables associated with the intraoperative unresectability of HCCA. A nomogram was created to calculate unresectability. A calibration curve in both the training and validation cohorts supported the power of the nomogram. To the best of our knowledge, this is the first nomogram for predicting the intraoperative unresectability of HCCA, and it can objectively assist doctors and patients in making the optimal choice for treatment.

## Methods

### Patient selection

**Fig 1:**
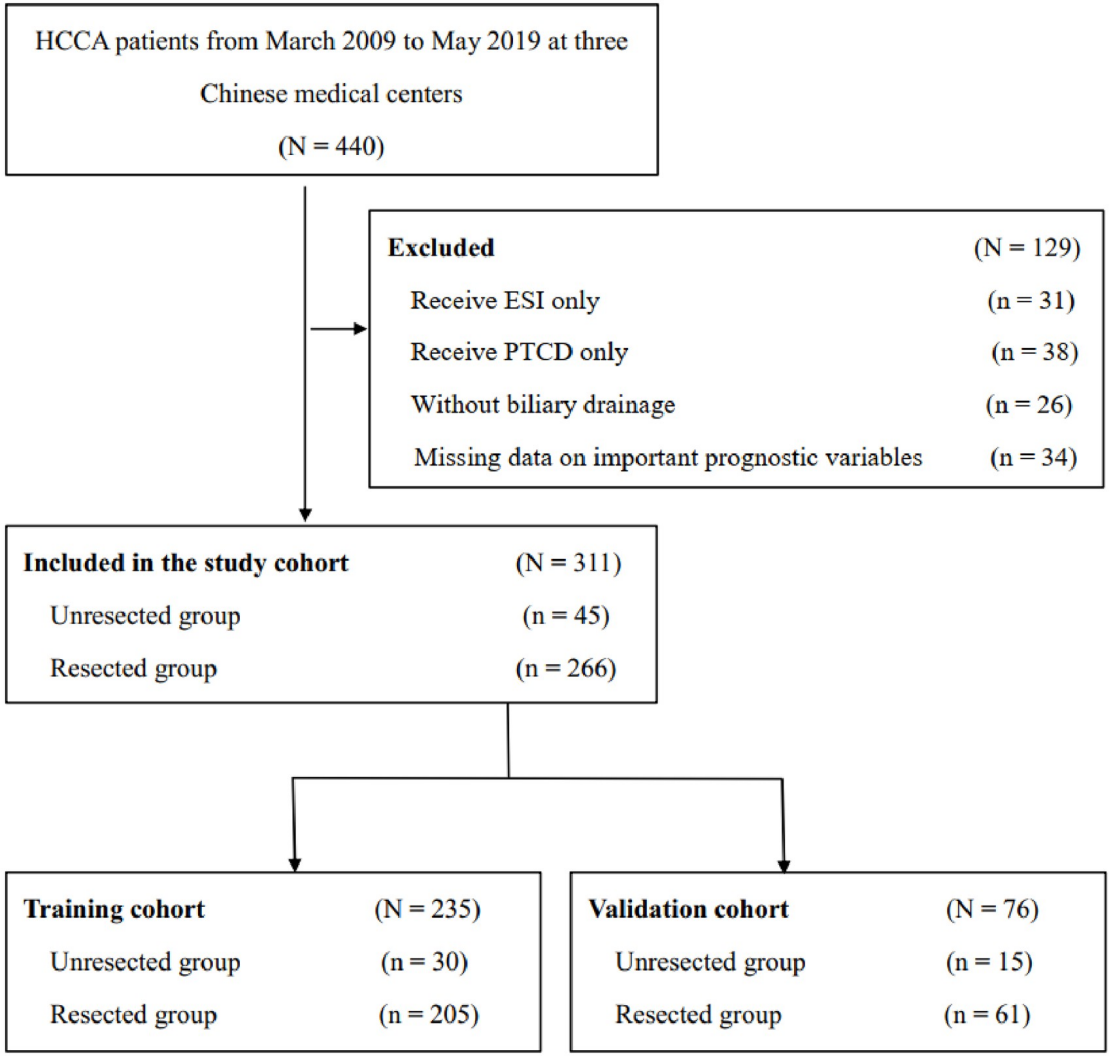
Selection of the study population. HCCA, hilar cholangiocarcinoma. ESI, endoscopic stent implantation. PTCD, percutaneous transhepatic cholangial drainage.

Four hundred and forty HCCA participants from three hospitals (Southwest Hospital, Sichuan Academy of Medical Sciences & Sichuan Provincial People’s Hospital and Qinghai University Affiliated Hospital) were included (Fig 1). Metastasis in the liver or other organs were excluded. The resected group was defined as patients receiving radical resection and negative incision margin was confirmed by pathology. Patients with high bilirubin level were routinely received biliary drainage and patients waiting for surgery usually received percutaneous transhepatic cholangial drainage (PTCD). The tumors in patients of the unresected group remained in situ, and they had routinely undergone biliary drainage for bilioenteric anastomosis during surgery or endoscopic stent implantation (ESI) postoperatively. Three hundred and eleven patients who had undergone exploration and 31 ESI patients were enrolled to compare the cost, surgery time and LOS. Patients who had undergone surgery were analyzed further. The pathological diagnosis of each patient was confirmed. The training cohort comprised patients from 2009 to 2016 while the validation cohort comprised patients from 2017 to 2019. The authors were accountable for all aspects of the work in ensuring that questions related to the accuracy or integrity of any part of the work were appropriately investigated and resolved. The study was conducted in accordance with the Declaration of Helsinki. This study was approved by Ethics Committee of the First Affiliated Hospital of Army Medical University, PLA. Approval number is: KY2021129. As this is a retrospective study and no private information of patients is enrolled, the form of consent was waived.

### Preoperative variables

**Fig 2:**
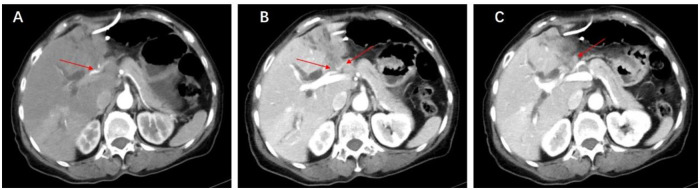
Illustration of hepatic artery, portal vein invasion and lymph node metastasis. A. Invasion of right hepatic artery (red arrow). B. Invasion of left portal vein and left hepatic artery (red arrow). C. Lymph node metastasis (red arrow).

The blood test results were obtained three days before surgery. Radiological examinations were performed within one month before surgery. For each HCCA patients, we routinely performed thin slice scan of CT (1.5mm or 1.25mm each slice) to obtain more details. In addition, Magnetic resonance cholangiopancreatography (MRCP) was administrated to show biliary tree and determine Bismuth type and make surgical decision. Definitive radiological findings included BC classification, organ or peritoneum metastasis, distant lymph node (LN) metastasis and locally advanced tumors. Pathological features included atrophy (future liver remnant), the tumor diameter, portal vein (PV) invasion and hepatic artery (HA) assessed via radiological examination (Fig 2). Both radiologists and surgeons dedicated hepatobiliary diseases. All patients were discussed among experienced hepatobiliary doctors. Serum parameters included hepatitis B virus (HBV), hemoglobin, total bilirubin, prothrombin time (PT), albumin, alanine aminotransferase (ALT), aspartate aminotransferase (AST) and tumor biomarkers (CA19-9, CA242, CA125 and CEA). Furthermore, variables such as age, sex, the American Society of Anesthesiologists (ASA) score, body mass index (BMI), malnutrition, biliary drainage, total cost, surgery time and LOS were included. The Nutritional Risk Screening (NRS) 2002 is the most common nutritional risk screening tool used clinically [12], and malnutrition was defined as an NRS 2002 score >2 points. Total costs were adjusted to the 2018 Chinese yuan (RMB) using the Consumer Price Index (CPI).

### Definition of unresectability

According to both Chinese and Japanese guidelines [13], we adopted the following criteria of unresectability and performed exploration in the following order:

1. Organ or peritoneum metastasis
  Organ metastasis included extrahepatic metastasis and metastasis in the remnant liver.
2. Distant lymph node metastasis
  In addition to lymph nodes (LNs) (No. 12) in the hepatoduodenal ligament, others along the common hepatic artery (No. 8a) or around the pancreas head (No. 13) were categorized as regional LNs and could be completely eliminated. Only distant LNs (para-aortic or further) were contraindications for surgical resection.
3. Locally advanced tumor (vascular involvement or extended bile duct invasion)
  Vascular involvement-related unresectability referred to involvement of the contralateral or main trunk of the HA or PV and the inability to be reconstructed.
  Extended bile duct invasion referred to the inability to restore biliary continuity with adequate remnant hepatic parenchyma.

### Statistical analysis

Statistical analyses were mainly performed using SPSS software version 25.0 (SPSS, Chicago, IL, USA). Continuous variables were presented as means ± standard deviation or medians (IQR). Categorical variables were presented as numbers (n) and proportions (%). Student’s t-test or the Mann−Whitney U test was used to compare continuous variables. χ2 test or Fisher’s exact test was used to compare categorical variables. Univariate and multivariable logistic regression analyses were used to identify the independent risk factors for intraoperative unresectability. Variables with P < 0.10 in univariable analysis were selected for further multivariable logistic regression analysis. A nomogram was established for the unresectability rate using the *rms* package with R Version 1.1.463 (http://www.r-project.org/). The final model was selected by the backward step-down method according to the Akaike information criterion [14]. Bootstrapping with 1,000 resamples was used to reduce bias. The internal validation of the nomogram was visualized using a calibration curve to depict the agreement between nomogram-predicted and actual unresectability rates. The performance of the nomogram was also measured by the concordance index (C-index). P < 0.05 was defined as statistically significant in all analyses.

## Results

### Patient characteristics

Three hundred and eleven patients who had undergone surgery and 31 patients who had undergone ESI were evaluated for cost, surgery time and LOS (Table 1). Surprisingly, we found no difference in the total cost (132343.2±53037.9 *vs*. 143669.8±71802.9 RMB; p = 0.359) or LOS (20.6±8.4 *vs*. 21.8±10.9 days; p = 0.439) between the unresected and resected groups. However, unresected patients had a shorter surgery time than resected patients (420.3±139.1 *vs*. 516.3±149.7 min; p = 0.001). Comparing the unresected and ESI groups, the former had a significantly higher cost (132343.2±53037.9 *vs*. 39284.1±10279.1 RMB; p<0.001), longer surgery time (20.6±8.4 *vs*. 12.1±4.7 days; p<0.001) and longer LOS (420.3±139.1 *vs*. 42.8±13.2 min; p<0.001).

**Table 1:** Comparison of total cost, surgery time and hospital stay between resected group or endoscopic stent implantation (ESI) group with unresected group.

	Unresected group	Resected group	*P-*value	ESI group	*P-*value
**Total cost (RMB)**	132343.2±53037.9	143669.8±71802.9	0.359	39284.1±10279.1	<0.001
**Surgery time (min)**	420.3±139.1	516.3±149.7	0.001	42.8±13.2	<0.001
**Length of stay (day)**	20.6±8.4	21.8±10.9	0.439	12.1±4.7	<0.001

*Notes*: ESI, endoscopic stent implantation; P-value was calculated by Student’s t test when resected or ESI group compared with unresected group, respectively.

Among the excluded patients, 69 had received only biliary drainage for ESI (31 patients) or percutaneous transhepatic cholangial drainage (PTCD, 38 patients). Twenty-six patients did not receive biliary drainage. Another 34 patients were excluded because of missing data. Ultimately, the data of 311 patients who had undergone surgical exploration were analyzed further (Fig 1). Among the 311 patients, 45 (14.7%) were judged as unresectable according to the criteria of unresectability. Two hundred and thirty-five patients from 2009 to 2016 comprised the training cohort (30 unresected and 205 resected). Seventy-six patients from 2017 to 2019 comprised the validation cohort (15 unresected and 61 resected). Hepatopancreatoduodenectomy was performed in 6 patients to achieve R0 resection of the distal bile duct following a reported recommendation [15]. For radical surgery, hepatectomy, lymphadenectomy and bilioenteric anastomosis were routinely performed.

The patients’ preoperative characteristics were compared between the unresected and resected groups (Table 2). When classified by the Bismuth-Corlette system, type III tumors comprised most cases (170, 54.7%), while types I&II (69, 22.1%) and type IV (72, 23.2%) tumors were shown. BC type IV patients had a lower resectability rate (45/72, 62.5%) than BC type I&II (65/69, 95.5%, p<0.001) or BC type III (156/170, 92.7%, p<0.001) patients. No difference was found between BC type I&II and BC type III patients (p = 0.46).

**Table 2:** Comparisons of preoperative variables between the unresected and resected groups.

Variables	Total (N = 311)	Unresected Group (N = 45)	Resected Group (N = 266)	*P-*value
Age, years	57.7 ± 10.0	58.9 ± 11.1	57.5 ± 9.8	0.411
Sex, male	178 (57.2)	21 (46.7)	157 (59.0)	0.142
BMI	22.1 ± 2.9	22.3 ± 3.1	22.1 ± 2.9	0.674
ASA score > 2	23 (7.4)	5 (11.1)	18 (6.8)	0.969
Malnutrition	98 (31.5)	12 (26.7)	86 (32.3)	0.473
HBV (+)	22 (7.1)	2 (4.4)	20 (7.5)	0.454
Cirrhosis	11 (3.5)	3 (6.7)	8 (3.0)	0.416
Preoperative biliary drainage	105 (33.8)	19 (42.2)	86 (32.3)	0.200
Preoperative hemoglobin, g/L	120.4 ± 21.8	118.0 ± 16.0	120.8 ± 22.6	0.589
Preoperative PT, s	11.3 ± 1.3	11.2 ± 0.9	11.3 ± 1.3	0.772
Preoperative bilirubin, μmol/L	166.48 ± 115.59	205.63 ± 117.56	159.9 ± 114.2	0.013
Preoperative albumin, g/L	36.7 ± 4.4	38.4 ± 4.6	39.9 ± 3.7	0.277
Preoperative ALT, U/L	77.1 (105.9)	75.0 (126.1)	70.0 (103.4)	0.995
Preoperative AST, U/L	77.0 (84.8)	69.6 (136.2)	67.0 (72.7)	0.468
CA19-9, U/ml	202.4 (429.7)	335.4 (484.6)	186.1 (396.2)	0.143
CA242, U/ml	18.29 (74.18)	40.57 (97.47)	18.1 (56.09)	0.245
CA125, U/ml	15.80 (20.30)	20.00 (23.60)	14.99 (18.33)	0.036
CEA, μg/L	2.50 (2.80)	2.76 (2.32)	2.47 (2.83)	0.709
Bismuth-Corlette classification				< 0.001
I&II	69 (22.1)	4 (8.9)	65 (24.4)	
III	170 (54.7)	14 (31.1)	156 (58.6)	
IV	72 (23.2)	27 (60.0)	45 (16.9)	
Liver atrophy	55 (17.7)	10 (22.2)	45 (16.9)	0.006
Tumor diameter, cm	3.0 (1.7)	3.0 (1.2)	3.0 (1.9)	0.984
Lymph node metastasis	67 (21.5)	18 (40.0)	49 (18.4)	0.001
Portal vein invasion	101 (32.4)	18 (40.0)	83 (31.2)	0.050
Hepatic artery invasion	80 (25.7)	23 (51.1)	57 (21.4)	< 0.001

*Notes*: Continuous variables are presented as mean ± standard deviation or median (IQR). Categorical variables are presented as number (percentage). BMI, Body Mass Index; ASA, American Society of Anesthesiologists; HBV, hepatitis B virus; PT, prothrombin time; ALT, alanine aminotransferase; AST, aspartate transaminase.

### Description of unresectable patients

According to the criteria of unresectability, 45 patients were defined as having unresectable tumors after exploration (Table 3). Among the patients, 15 had distant metastasis, including organ or peritoneum metastasis (35.6%), and 8 had distant LN metastasis (17.8%). Locally advanced tumors showed extended vascular involvement (12, 26.7%) and bile duct invasion (9, 20.0%).

**Table 3:** Reasons of unresectability judgement after exploration.

Reason	Cases (%)
Organ or peritoneum metastasis	16 (35.6)
Distant LN metastasis	8 (17.8)
Extended vascular involvement	12 (26.7)
Extended bile duct invasion	9 (20.0)
Total	45

### Independent risk factors for unresectability in the training cohort

Logistic regression was performed to identify risk factors (Table 4). Preoperative bilirubin (p = 0.070), CA125 (p<0.001), BC type IV (p<0.001), liver atrophy (p = 0.009), lymph node metastasis (p<0.001) and HA invasion (p<0.001) showed p values <0.1 in univariable regression and were entered into multivariable regression. The 95% confidence interval (CI) and odds ratio (OR) were calculated. Finally, multivariable analyses demonstrated that CA125 (OR, 1.026; 95% CI, 1.012–1.041; p<0.001), BC type IV (OR, 6.090; 95% CI, 1.758–21.100; p = 0.004), lymph node metastasis (OR, 10.088; 95% CI, 2.820–36.082; p<0.001) and HA invasion (OR, 7.194; 95% CI, 2.166–23.888; p = 0.001) were independent risk factors for intraoperative unresectability.

**Table 4:** Univariable and multivariable logistic regression analyses of training cohort predicting intraoperative unresectability.

Variables	Univariable *P*-value*	Multivariable *P*-value	Multivariable OR (95%CI)
Age	0.877		
Sex (Male vs female)	0.336		
BMI	0.906		
ASA score (> 2 vs < 3)	0.815		
Malnutrition	0.945		
Preoperative biliary drainage	0.440		
Preoperative hemoglobin	0.451		
Preoperative bilirubin	0.070	0.547	
Preoperative albumin	0.702		
Preoperative ALT	0.561		
Preoperative AST	0.295		
CA19-9	0.405		
CA242	0.589		
CA125	< 0.001	< 0.001	1.026 (1.012–1.041)
CEA	0.544		
BC type IV	< 0.001	0.004	6.090 (1.758–21.100)
Liver atrophy	0.374		
Tumor diameter	0.547		
Lymph node metastasis	< 0.001	< 0.001	10.088 (2.820–36.082)
Portal vein invasion	0.114		
Hepatic artery invasion	< 0.001	0.001	7.194 (2.166–23.888)

*Notes*:

* Those variables found significant at P < 0.1 in univariable analyses were entered into multivariable regression analyses. BMI, Body Mass Index; ASA, American Society of Anesthesiologists; HBV, hepatitis B virus; PT, prothrombin time; ALT, alanine aminotransferase; AST, aspartate transaminase.

### Nomogram predicting the unresectability of HCCA

**Fig 3:**
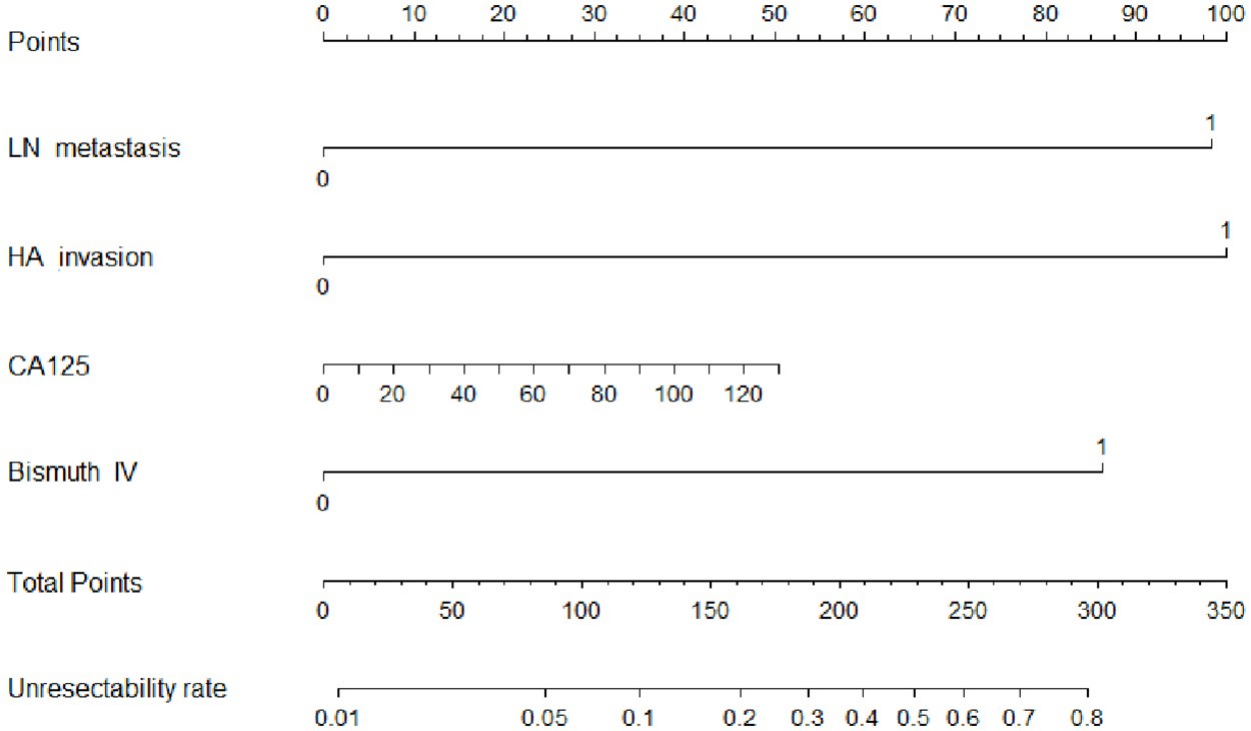
Nomogram predicting intraoperative unresectability of HCCA in training cohort.

**Fig 4:**
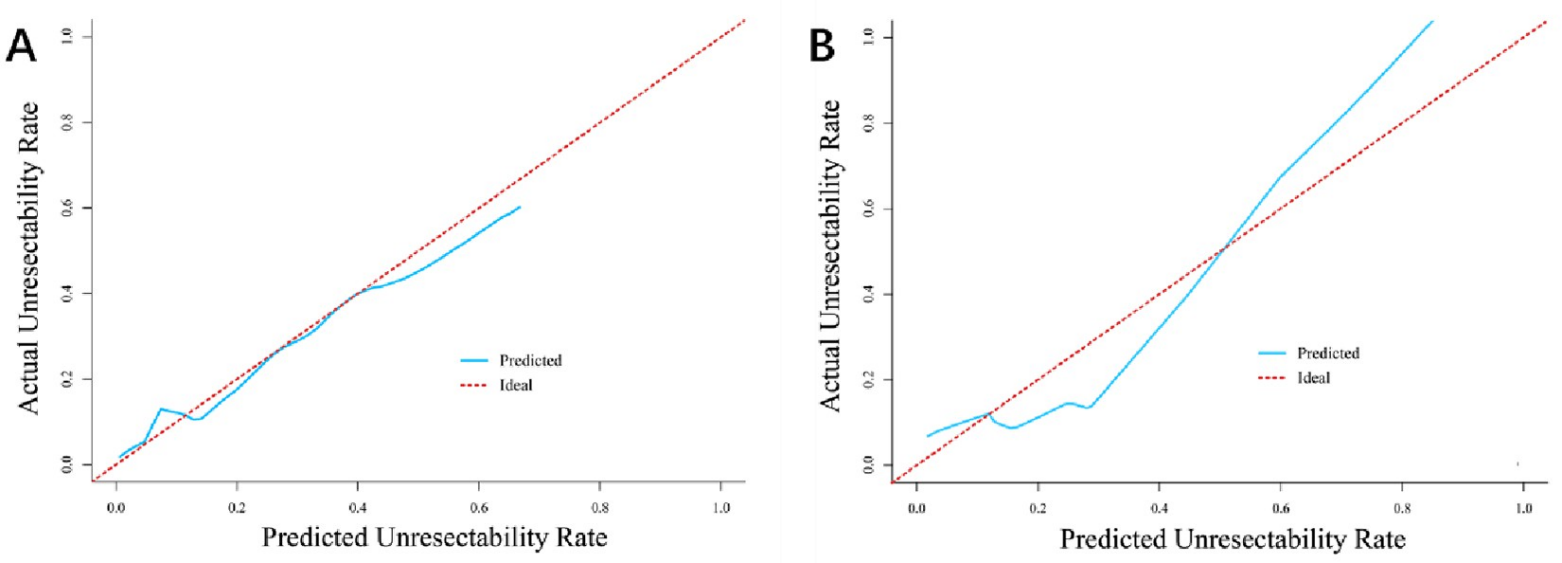
The calibration curve for predicting unresectability rate of training (A) and validation (B) cohort.

The independent factors in the training cohort from multivariable logistic regression were integrated into the nomogram and are shown in Fig 3. A calibration curve comparing the predicted and actual unresectability rates of both the training and validation cohorts was created and showed satisfactory agreement (Fig 4). The C-index values of predicting the unresectability rate of the training and validation cohorts were 0.900 (95% CI, 0.835–0.966) and 0.829 (95% CI, 0.546–0.902), respectively.

## Discussion

Preoperative evaluation does not completely avoid nontherapeutic surgical exploration [16]. When complete resection cannot be performed during surgical exploration, biliary drainage is only applied for cases such as bilioenteric anastomosis or postoperative ESI. The survival of these unresected patients is similar to that of patients without exploration. Their natural lifespan is approximately 5–13 months, which is significantly lower than that of resected patients [17, 18]. Moreover, we found that the unresected HCCA patients did not have lower hospitalization costs or a shorter LOS than resected patients but more pain from the incision and mental stress than endoscopic drainage patients. This finding reveals the price of futile surgical exploration and presents the importance of the preoperative evaluation of intraoperative unresectability. By analyzing preoperative clinicopathological features, we identified that risk factors for intraoperative unresectability include BC IV type, lymph node metastasis, HA involvement and CA125. A nomogram was built to directly predict the unresectability rate, providing an objective reference to make preoperative choices.

The main reasons for unresectability were organ or peritoneum metastasis, lymph node metastasis, extended vascular involvement and extended bile duct invasion, which are listed in Table 3 [3, 10]. Staging systems such as the MSKCC system are mostly based on these features [5]. However, the influence of each factor on the outcome was not quantified. Additionally, some studies have reported HA involvement and the nodal status as predictors of resectability. This conclusion was derived from univariate logistic regression, and the p value for the regression was not disclosed [4], making the result questionable. Furthermore, main or bilateral HA or PV invasion was previously believed to be strongly associated with unresectability [10]. With the development of surgical and anesthetic techniques, more resections can be achieved than before, and the criteria of intraoperative unresectability may have changed. Furthermore, PV invasion can be approached by reconstruction with or without artificial vessels. As far as we know, staging laparoscopy is mainly performed when distant metastasis cannot be excluded. When distant metastasis excluded, surgical exploration in porta hepatis was usually attempted in our practice to achieve complete resection even with vessel reconstruction. With the development of radiologic exams, most patients with distant metastasis can be recognized and this reduced stating laparoscopy in practice.

Thus, reassessing the risk factors and assigning the corresponding impact of each variable could comprehensively indicate the unresectability of HCCA. Regression analysis provided the degree of impact of each variable, as measured by the odds ratio, and a nomogram was ultimately constructed. Nomograms for surgical resectability are rare and have only been recently reported for gallbladder cancer with preoperative CT features to predict the resection margin [19]. A nomogram predicting the prognosis of HCCA has been reported [20, 21], but none for HCCA intraoperative resectability exists. To the best of our knowledge, this is the first nomogram for predicting HCCA unresectability. This tool could supply clinicians with visualized possibilities to make objective choices for individuals and avoid pointless surgery. For patients with high risk calculated by the nomogram, additional imaging such as PET/CT and diagnostic laparoscopy may be necessary when making surgical decisions.

The criteria for resectability varied to some extent in different centers at different times [5, 10, 22, 23]. Among them, metastasis (organs or distant lymph nodes), extended bile duct invasion and PV invasion were the basic factors for evaluating resectability, while HA reconstruction was not regularly suggested for surgery. The American and European guidelines were relatively conservative [16, 24]. The primary criteria for resectability were biliary reconstruction options and sufficient remnant liver, and vascular reconstruction was not routinely recommended. Even portal vein reconstruction was only performed in highly selected patients. However, medical centers, including ours in East Asia (particularly China and Japan), have performed aggressive surgery to achieve en bloc resection, including extended hepatectomy, extensive lymphadenectomy and vascular reconstruction of both PV and HA, resulting in a better prognosis [16, 25–27]. Additionally, as mentioned above, regional lymph nodes included No. 8a, 12 and 13, while American and European guidelines only recommend LNs in the hepatoduodenal ligament (No. 12).

As mostly recommended, hepatectomy for HCCA is based on bile duct invasion according to the BC classification, which correlates with resectability and metastasis [1, 4]. Bile duct-based unresectability can be assessed preoperatively by calculating the future liver volume according to the anticipated resection liver area. Biliary drainage is frequently performed to reduce liver failure [28]. During surgical exploration, extended bile duct invasion, particularly in BC type IV patients, could attenuate the resectability rate. The resection rate of all BC types was less than 50%, and the lowest rate was for BC type IV (14.0%) [3]. In recent years, surgical performance and the R0 resection rate have been notably increased. BC type IV HCCA could even achieve a resectability of 62% (254 of 411 cases) [29]. In our study, the resectability rate of BC type IV tumors (67.1%) was lower than that of BC types I&II (95.5%) or BC type III tumors (92.7%). This finding supported that a BC type IV classification may correlate with the unresectability of HCCA.

PV invasion was not a contraindication in our criteria, and reconstruction of the PV was performed regularly. This finding agreed with the multivariable regression result that PV invasion is not a risk factor for unresectability. Distant lymph node metastasis and HA (particularly contralateral) invasion are the main uncertain parameters of the preoperative assessment and the reasons for unresectability during exploration. As mentioned above, our study confirmed that intraoperative unresectability is significantly correlated with HA invasion and distant lymph node metastasis. HA reconstruction remains controversial, although it improves complete resection. Patients who had undergone HA reconstruction showed worse outcomes (5-year disease-free survival, 22.3%) and higher lymph node metastasis rates (59%) than those without HA reconstruction. However, the survival of these patients still exceeds that of unresected participants, with a median survival of approximately 1 year [17, 18]. Consequently, we still recommend HA reconstruction in experienced centers and for selected patients.

In addition to having diagnostic value, tumor biomarkers are correlated with recurrence, treatment response, lymph node metastasis and even resectability in multiple cancers [30–32]. Preoperative assessments of CEA, CA19-9 and CA125 are recommended in HCCA patients [1, 33]; CA19-9 and CA125 are risk factors for the unresectability of HCCA [33]. CA19-9 was also included in the reported HCCA staging system [34]. Additionally, the ability of CA125 to predict pancreatic cancer resectability was superior to that of CA19-9 [35]. CA125 has also been proven to indicate the resectability of cholangiocarcinoma with both a sensitivity and specificity beyond 70% and an area under the receiver operating characteristic (ROC) curve of 0.81 [9]. However, only 50 samples were enrolled in the analysis; thus, this conclusion may not be convincing. In our study, we clarified that CA125 is an independent risk factor indicating the unresectability of HCCA, while CA19-9, CA242 and CEA are not. This finding provides additional criteria of tumor malignancy when considering surgical exploration and prognosis, in addition to the pathological features.

As previous studies proved, the tumor diameter was not a risk factor for prognosis, while vascular involvement and distant metastasis were highly correlated with the malignancy degree and survival [18, 21, 36]. Similarly, the tumor diameter was not correlated with unresectability in this study. Tumor volume expansion in situ may enhance surgical difficulty; however, resection can mostly be achieved by skilled surgeons. Specifically, borderline resectability is difficult to evaluate, and radiological examination cannot supply sufficient convincing predictions. In clinical practice, surgical exploration is still recommended for selected patients with potential resectability. Unfortunately, the unresectability rate ranges from approximately 42%-55% [4, 5, 22, 37]. A previous study revealed that the median waiting time for HCCA surgery was 74 days, which did not impact resectability, metastasis, tumor stage or survival [38]. Consistent with this conclusion, we suggest evaluating borderline HCCA patients receiving preoperative neoadjuvant therapy, similar to the evaluation process for pancreatic cancer patients [39]. These noninvasive methods could downstage and evaluate the sensitivity of chemotherapy, which assists in evaluating surgical necessity and reducing the number of senseless exploration procedures. Additionally, portal vein embolism (PVE) or associating liver partition and portal vein ligation for staged hepatectomy (ALPPS) was another option to achieve resectability through increasing the volume of the remnant liver [40, 41]. Beyond guideline recommendations, a cancer-positive periaortic node was not a total contraindication of resection [13, 42].

Hyperbilirubinemia was observed in all 45 unresectable patients. All the patients who received biliary drainage had undergone either intraoperative bilioenteric anastomosis or postoperative ESI. Only a small proportion of patients had received only PTCD. Intraoperative exploration and bilioenteric anastomosis were the main reasons for the mean surgery time of more than 7 hours. Moreover, most patients remained in the hospital for recovery (nutrition and incision healing) because we did not have community resources for postoperative recovery. For some patients, complications including infections also increased the cost and LOS. Considering these factors, the total cost and LOS were unlikely different between the resected and unresected groups.

This study has several limitations. First, this was a retrospective study, and some inherent limitations cannot be avoided. Some data could not be rerecorded. Second, all the patients were recruited from 2009 to 2019, but the radiological examinations and operation skills were evaluated within a decade. Biases in the radiological assessments and resectability evaluations among patients in different years exist. Third, slight deviations might have occurred in the preoperative radiological assessments and unresectability criteria among the three centers. Finally, the sample size was not sufficiently large and grouping deteriorated the lack of samples. Further investigation with more samples is warranted.

## Supporting information

S1 Data (R)
